# Assessment of Factors Affecting Fish Production and Marketing in Gambella Region, Ethiopia

**DOI:** 10.1155/2020/5260693

**Published:** 2020-06-16

**Authors:** Gatriay Tut Deng

**Affiliations:** Zoological Science, Mekdela Amba University, P.O. Box 32, Tulawlia, Ethiopia

## Abstract

Fish production has been practiced since ancient Egypt and China. It has become a fast-growing agricultural sector that provides animal protein for most people globally. While China is the leading country in the world, Egypt and Nigeria stand on the top in Africa. The overall fishery production potential of Ethiopian water bodies is estimated to be 94,500 tons per year, while the actual production is 38,370 tons. Despite more water bodies and more fish diversity in the region, fish production and marketing is very low. This review is undertaken to assess the main factors affecting fish production and the marketing chain in Gambella region. Despite the known water and fish potential of the region, fish production is very low. Major factors contributing to a reduction in fish production in the region include inefficient fishing gears, poor transportation access, poor postharvest handling, low price at the landing site, and improper market place. Drying is the predominant postharvest technique and fishing methods are of a subsistence basis. All the fishing activities take place in the natural environment, and aquaculture is not yet established. Enough modern and efficient gears need to be made available. Other modern postharvest handling techniques need to be introduced to ensure a longer shelf life of fish after harvest. Infrastructures need to be constructed to access all water bodies in the region. Because the region has such water resource potential and incredible fish species diversity, the aquaculture needs to be established and popularized. Traceability needs to be adopted in the region to prevent food-borne diseases. Based on this paper, the government and other stakeholders could develop policy considering the issue of the fishery status of the region.

## 1. Introduction

Fish farming has been practiced in different parts of the world, particularly East Asia, China, Europe, Canada, Africa, and developing countries like Nigeria [1]. It has been in practice since the ancient civilization of Egypt and China. The fisheries sector remains an important source of food, nutrition, income, and livelihoods for hundreds of millions of people around the world [2].

With more focus on the nutritional value of food commodities, fish is acknowledged as a major nutrient-rich animal food source for a significant proportion of the nutritionally vulnerable people, overshadowing that of most of the terrestrial animal foods. In the last decade, of the 30 countries where fish contribute more than one-third of the total animal protein supply, 22 are Low Income and Food Deficient countries (LIFDCs) [3]. Furthermore, in addition to animal protein, fish contain unique long-chain polyunsaturated fatty acids (LC-PUFAs) and highly bioavailable essential micronutrients, vitamins D and B, and minerals (calcium, phosphorus, iodine, zinc, iron, and selenium). These compounds, often not readily available elsewhere in diets, have beneficial effects for adult health and child cognitive development [4].

The contribution of fishery activities to national economies is multifaceted. In addition to supplying food, the fishery sector contributes to gross domestic product (GDP), provides livelihoods for fishers and processors, is a source of hard currency, and boosts government revenues through fisheries agreements and taxes [5].

In boosting the economy, world per capita fish supply reached a record high of 20 kg in 2014 [2]. This impressive development has been driven by a combination of population growth, rising incomes, and urbanization. It has been facilitated by the strong expansion of fish production and more efficient distribution channels. Although annual per capita consumption of fish has grown steadily in developing regions (from 5.2 kg in 1961 to 18.8 kg in 2013) and in low income food-deficit countries (LIFDCs) (from 3.5 to 7.6 kg), it is still considerably lower than that in more developed regions [2]. China has been the leading among the developed nations in its fish production, particularly from aquaculture with per capita apparent fish consumption of about 35.1 kg per year in 2010 [5]. While the annual per capita fish supply in the rest of the world was about 15.4 kg in 2010. In developing countries, fish consumption tends to be based on locally and seasonally available products, with supply driving the fish chain. However, fuelled by rising domestic income and wealth, consumers in emerging economies are experiencing a diversification of the types of fish available owing to an increase in fishery imports.

Aquaculture is still developing in Africa and is mostly concentrated in a few countries but already produces an estimated value of almost US$3 billion per year [5]. The value added by the sector as a whole in the last decade was estimated at more than US$24 billion, 1.26 percent of the GDP of all African countries. Despite the fact that the industry is in its infant stage, the continent is working really hard in getting use of the sector in fighting poverty. In line with this, studies reported that the fisheries sector as a whole employs 12.3 million people as full-time fishers or full-time and part-time processors, representing 2.1 percent of Africa's population of between 15 and 64 years old. Expanding on what the figure indicates, at the country level, Nigeria ranks first with almost 2 million people engaged in the fisheries and aquaculture sector, followed by Morocco (almost 1.4 million) and Uganda (almost 1 million) [5].

It is wise to put forth the distinction between these two capture fisheries aquatic ecosystems, marine and inland ecosystems, as they influence the fish species being targeted. The inland fishery of Africa is estimated to contribute to about 2.1 million tonnes of fish per year. It represents 24% of the total world fish production from inland water bodies [6]. In Ethiopia, fish comes exclusively from inland water bodies including lakes, rivers, streams, reservoirs, and substantial wetlands that are of great socioeconomic, ecological, and scientific importance [7–9]. Fishing has been the main source of protein supply for many Ethiopians, particularly for those who are residing in close proximity to major water bodies such as Lake Tana, Ziway, Awassa, Chamo, and Baro River [5, 9].

The overall potential yield of fish in Ethiopian water bodies is estimated to be 94,500 tons per year on average [9]. Water bodies located in the Rift Valley show signs of overexploitation, whereas those located in remote areas with poor infrastructure which make up the majority remain underutilized [8, 9]. Hence, the existing role of the fishery is insignificant in the country's overall economy because the fishery sector in the country is far below its potential [10]. The current production is still far below the estimated potential yield, which suggests the possibility for further expansion of the fishery.

As the presence of sufficient freshwater bodies may signify indirect fish availability, Gambella is endowed with several inland water resources including rivers, lakes, reservoirs, ponds, and huge floodplain areas but contributed far less to national fish production and marketing. From the literature, Gambella is the region with the most diverse fish species in its water bodies but rarely reported for its fish production and marketing. Therefore, this review is carried out to assess the major factors which could possibly contribute to the low fish production and how the fish market is oriented in the region.

## 2. Fish Demand and Delivery Pattern

Fish consumption varies greatly depending on the quality and quantity of fish supply available for consumption. Ethiopians have no tradition of consuming large quantities of fish, rather highly committed to rearing and consuming farm animals. The fact that most Ethiopians are not actively involved in fish consumption may also be a reason for them to have little participation in fish production. It is true that when a product is no longer demanded by the consumers (customers), the trader would not supply it anymore and shift to other products in demand. Knowing the nutritional and economic value of fish, for how long will the tradition of low fish consumption and demand continue?

In religious perspectives, the Ethiopian Orthodox Church undergoes several fasting periods when they refrain from meat consumption and consider fish as good enough to be consumed [11]. In those occasions, people developed the culture of fish consumption in part of the country where fish is not part of their daily meals. By taking advantage of these fasting periods, a large amount of fish should be produced and supplied in order to fill the gap of high fish demand which could lead to adopting fish consumption tradition in parts of the country with low fish consumption. On the other hand, fish consumption tradition is highly practiced in a limited part of the country where they use it as part of their regular meals like in the Gambella region, including in the period of fasting. Increasing fish demand and shortage of supply result in rising fish prices. So, this escalates the affinity for fish to be a luxury product consumed by rich people. Gordon et al. [12] also suggested that there is inadequate evidence that higher income groups may be a considerable source of the increase in fish demand despite population increase, particularly in a big city and a general increase of demand.

Regardless of the big rivers and lakes found in the country, a Baro-Akobo basin is the most diverse in terms of fish diversity and its floodplain is the largest one in the country. Even though Baro River is recorded as the most diverse in fish species throughout the country, its contribution to national fish production is less documented. Although local people exploit the river system on a subsistence basis, they utilize it to fulfill their daily food requirements. Getahun [13] reported that the most locally important commercial fish species of this river system are *Oreochromis niloticus*, *Clarias* sp., *Polypterus bichir, Heterotis niloticus, Gymnarchus niloticus*, *Malapterurus* sp., *Lates niloticus*, *Alestes* spp., *Hydrocynus* spp., *Mormyrops* spp., *Bagrus* spp., *Barbus* spp, and *Labeo horei*. Moreover, Hussien et al. [14] reported more than 19 commercially important fish species in the Gambella region (Table 1), but most of these species are not well supplied to the big cities where fish fetches relatively good price because of low and inefficient facilities.

Local fisher's cooperatives and fishers being the sole exploiters, Baro River, Gilo River, and some other smaller wetlands in the floodplain are the major places where fishing activities are largely intensive. The catch estimates from rivers around Gambella is 5,000 tons/year, whereas the actual fish production potential is about 12,000 tons/year [15]. In nations where fishery sectors produce millions of tonnes of catch like China, highly sophisticated fishing methods and equipment are utilized. In contrast, fishers in Ethiopia particularly, the Gambella region, use traditional fishing methods and equipment. As a result, the catch obtained from these fishers is far less below the actual fishery potential of the river system.

Fishing is generally in the subsistence scale in this river system where fishes and fish products are chiefly used for household consumption, whereas a small amount is sold at local markets to obtain additional income. The majority (64.6%) of the surveyed fisher respondents who have access to the market mentioned that their usual clients were consumers and few (35.4%) of the respondents sold their products to the retail shops and hotels [16]. Because of low fish production in this river system, the majority of the catch is tending to be sold in the local market where the fishers easily accessed. Most of the people that live in close proximity to the water bodies tend to meet most of their animal protein requirements through fish consumption. Since fishing in these areas is in a small scale, fishery practice and gears used are of a traditional basis.

## 3. Fish Production and Marketing Challenges

The amount of fishes produced in a certain water system is determined relative to the potential the water system has to produce fishes. The fish production potentials of the main rivers of the Baro-Akobo Basin alone (viz. Baro, Akobo, Gilo, Alwero) were estimated to be 3,720 tons per year [14]. However, currently, the annual production is reported to be about 380 ton per year that is still far below the expected maximum sustainable yield of the water system. This low level of fish production may be attributed to the traditional fishing methods and equipment being utilized by the fishers. The region has a huge diversity of fish species but the bulk of the catch and the fish markets are dominated by 19 genera and more than 20 species (ibid).

The efficiency and modernity of the fishing materials and equipment determine the production status of a particular water body. The fishers in the Gambella region use different and traditional gears with more than 15 types. The usage of these gears tends to vary with seasons, methods of fishing, and the materials they are made of. Some modern fishing gears such as seine nets, hook, and line and cast net were supplied by NGOs operating in the region and are being used by individuals and fisher cooperatives [14, 16]. But these gears were still far insufficient to accommodate all the fishers in need. The scarcity of improved fishing gears and inefficiency of existing traditional gears may have contributed to the low fish production in the region.

Gambella experiences two conspicuous seasons: the dry season and wet season. Since the region is located in the lowland floodplain of the country, there is a regular incident of the flood that can cover the whole floodplain and displace most local residents in the lower elevation in the wet season. Because of this flood, most activities become disrupted and put into a halt in the wet season, including fishing. The seasonal halt in fish production implies that the fishing activities are not much intensive, and people do not exclusively utilize fish resources. In contrast to the nations where fishing is used to secure a huge amount of funds, there is no what so-called seasonal close down in fishing.

**Figure 1:**
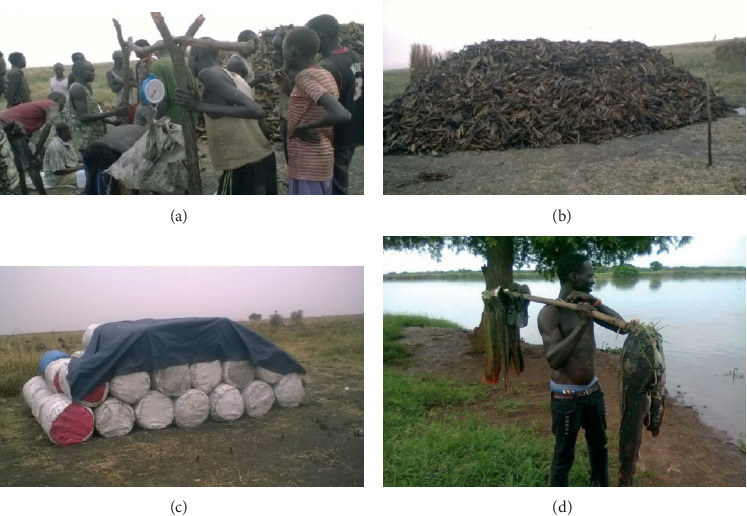
Fish marketing and transportation: fishers selling their dried fish to the local enterprises at the landing site (a); dry fish accumulated by the enterprises after being bought (b); dry fish are packed and made ready to be transported to big towns and cities (c); fisher carrying his catch to the nearby local market (d) (photos by Gatriay Tut).

The survey conducted by Abegaz et al. [14] interviewed fishers about the existing production constraints and ranked them according to their importance: inefficient fishing gear, lack of motorized boat service, poor transportation access, lack of value adding facilities, lack of fish handling facilities (like refrigerator), poor postharvest handling, crocodile attack, and gear theft problem. The fishers also added that they have faced marketing constraints like low price at the landing site, poor road access, high transportation cost, improper market place, lack of cold storage, and poor power supply (Figure 1). The abovementioned constraints are intensified because (i) there is no government intervention in promoting fishery activities, (ii) most NGOs operating in the region are not actively involved in supporting the fishery sector. Moreover, Dorgi and Gala [16] added that the majority (79.5%) of the surveyed fishery cooperatives responded that they do not have good prices for their products at the market. The price of the product is determined by its quality and preference by the customers. While local fishers are utilizing the traditional fishing system, the fish produced in this system is of low quality and results in low prices at the markets.

## 4. Fish Preservation and Transportation

Because of its high protein content, fish is one of the most perishable foods that can be spoiled easily if not properly preserved, particularly in tropical and subtropical climates [17]. Fish can still be subjected to a range of spoilages even if traditional preservation techniques have been used [18]. Fishes, like other commercial commodities, are needed to be transported from landing sites to places where they can be sold or utilized by the consumers. Because of their perishable nature, fish need very careful attention to maintain quality and avoid spoilage. Ethiopia mostly experiences a traditional stage in fish handling and preservation techniques. The fish captured are taken from on-site immediately and reach the market by traditional means of transportation without any preservation facilities to keep them fresh. The traditional means of fish preservation and transportation contributed much to the low quality of preserved fish and short lifespan of fish products. According to Dorgi and Gala [16], 64.8% of the surveyed fishery cooperatives in the Gambella region lack access to transportation services and the fishers used to carry their products to reach nearby market places on foot. Few of them with little access to transportation use bicycles and car transport. For the fishery sector to succeed in promoting the local and national economy, all stakeholders should participate in providing all the necessary services required to get the best out of the sector.

**Figure 2:**
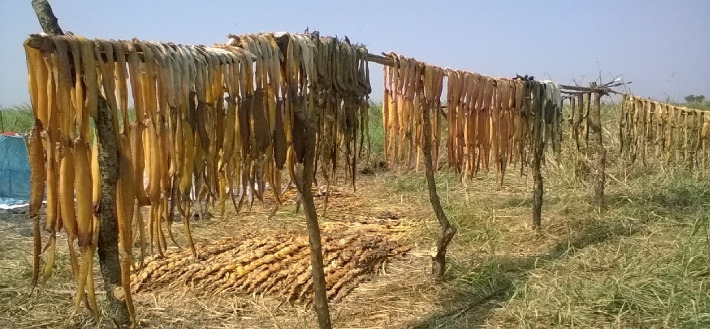
Fish fillets hanged for immediate sundry in Wagan wetland fishers' camp (photo by Gatriay Tut).

The most common form of fish preservation technique practiced in Gambella is drying. Sun drying is the simplest way of preserving fish and is practiced not only in Gambella but also in some Rift Valley lakes where fish are caught locally (Figure 2). Fishers remove gut, behead and fillet their catch, and expose them to sunlight for immediate dry up. The postharvest handling process of any products, including fish, is the procedure taken to ensure the quality of a product until handed over to the customer. This drying technique is mostly performed by local fishermen on remote fishing sites where they could spend some time before bringing their catches to the nearby markets [19]. The smoking technique is sparingly used, especially during the wet season when there is not enough sunlight. The smoked fish cannot be sold in the local market because of their low quality and can only be used for home consumption. The traditional posthandling techniques and transportation methods being used in the region contributed much in one way or the other to the low quality of fish resulting in low price in the market.

Deep freezing and cold rooms are also used in some cases by Fish Production and Marketing Enterprise centers in the Gambella region. Even though there are some modern preservation facilities like refrigerators for temporary fish collection, they do not have enough power supply to keep the facilities function properly. The problem is intense because most fish retail shops, fish collection, and storage facilities do not have backup diesel generators [20]. There is no report on salting as preservation methods in Gambella, but ICC [21] reported that some trials have been made at the Fishery Production and Marketing Enterprise collection center in Ziway for this technique.

## 5. Current Trends of Capture Fisheries and the Status of Aquaculture

Capture fishery involves the catching of valuable aquatic organisms from their natural environment. All the fish utilized in the country are collected from the wild by subsistence means. Though Gambella is known for its remarkable fish diversity in its water bodies, it contributes little to the national fish consumption. Most of the fishery resources consumed in the big cities and towns in Ethiopia are captured from the Rift Valley lakes (40%) and Lake Tana (50.2%) [22]. Subsistence capture fishing activities are performed in the Baro-Akobo river system and its tributaries in Gambella, mostly for local consumption [20]. Capture fisheries are the sole fishery strategy in the whole country, and its production is declining drastically because of several reasons. These include agricultural expansion, pollution, climate change, siltation, irrigation schemes, overfishing, and so on. Despite favorable physical and hydrographic conditions (suitable geographic relief, rich soil quality, good mean annual rainfall, and sufficient freshwater availability), aquaculture production is negligible in Ethiopia. Since the fishery experts understood the value of fishes, a fish campaign is underway where the local people as a whole are to be aware of the benefits of fishes and take part in fish farming.

So there is an urgent need to establish aquaculture in the country for farming valuable aquatic organisms under controlled artificial environments to replace the declining capture fishery. There is no report about Aquaculture in the Gambella region. Because of the region's water bodies and fish potential, it is in the mind of fisheries experts to establish aquaculture in the region. Therefore, Gambella is regarded as “the dreamland for aquaculture,”

## 6. Concept of Food Traceability, Importance, and Its Future Direction

Food safety has become a growing concern for citizens of many countries where they expect safe and nutritious foods. Today, food safety is a worldwide concern due to a number of food safety scandals. Outbreaks of diseases such as *Escherichia coli*, African swine fever, highly contagious diseases such as avian flu in poultry, bovine spongiform encephalopathy (BSE) and foot and mouth disease in livestock, presence of dioxin, and microorganisms like Salmonella, Norovirus, Campylobacter, Listeria, Clostridium [23], and Coronavirus (COVID-19) have resulted in heightened public and private attention to food attributes. Furthermore, because of globalization in which the world becomes a single village, large numbers of people embark on international travels and trade, which escalates these outbreaks to reach a pandemic extent. This important concept, “the traceability,” is less practiced in many low- and lower-middle-income countries (LMICs), including Ethiopia, where disease burdens caused by poor food supply chain are highest.

### 6.1. Importance of Traceability

For the better wellbeing of the people, a reliable traceability technique is needed to inspect all the food products, including fishes being supplied. Good traceability in food supply chains has the potential to reduce risks and costs associated with food-borne disease outbreaks, product recalls, maintaining consumer, or market confidence in product [24, 25]. In addition, electronic-based traceability increases production efficiency and also saves labor costs compared to paper-based systems [26, 27]. Another economic reason for adopting traceability systems is to eliminate liability risks associated with unsafe food problems [24] which may result in financial damage such as penalties, loss of trade, damage to reputation, or loss of brand name capital [25]. Good traceability systems decrease the probability of a supplier being found wrongly liable for a certain food safety problem and would give the opportunity to improve the overall level of food safety.

**Figure 3:**
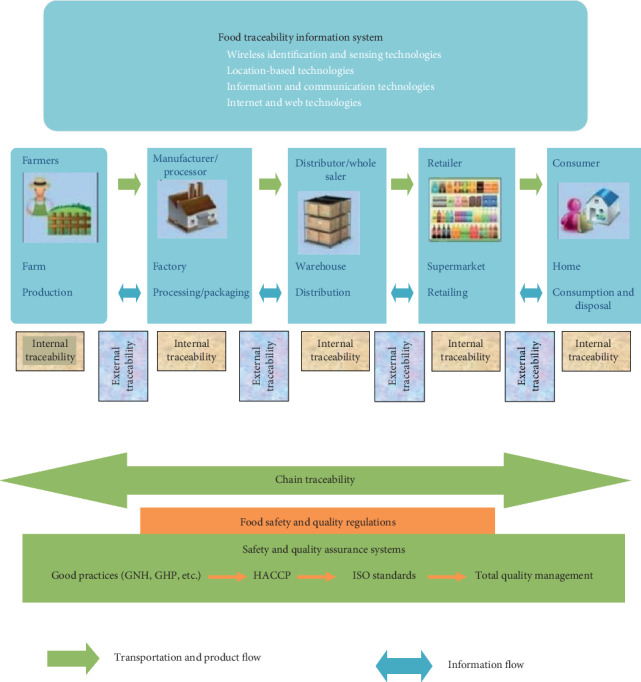
Traceability in a food supply chain: safety and quality perspectives [28].

Improving traceability at the supply chain level can potentially reduce the costs to downstream actors in monitoring the activities of upstream steps [24, 25]. Readily verifiable traceability information can lead to a reduction in information costs aimed towards consumers associated with quality verification [24]. The importance of traceability in food safety and quality maintenance in the food supply chain is illustrated in Figure 3.

### 6.2. Traceability Tools and Technology Solutions

Traceability implementation is a tedious task. Thus, it requires automated data collection to reduce much of the time and expenses required for data processing and maintenance. Gathering information for large operations manually is time-consuming and leads to risks of recording the information incorrectly. For example, errors occur in 36% of consumer packaged goods orders according to a study by the Grocery Manufacturers Association (GMA) in the United States [29]. Such errors lead to inventory inaccuracies and stock ruptures. Therefore, traceability initiatives rely on technologies to provide efficient, accurate ways to track and trace products and their movement across the supply chain. This includes technology for product identification, information capture, analysis, storage, and transmission of data as well as overall systems integration. Such systems include hardware such as measuring/sensing equipment, identification tags, and labels, with software. Data collection using tools such as barcode and Radio Frequency Identification Device (RFID) is exceptionally accurate (>99%) [30]. These tools scan, record product codes, lot numbers, invoice data, order numbers, and other information in less than a second.

Moreover, among many tools and techniques developed, [31] suggested that the PCR-DGGE technique is a viable method to meet both traceability and safety requirements for seafoods at the same time. This PCR-DGGE, a new molecular technique of traceability, is used to trace and discriminate the geographical origin of fish by analysis of DNA fragments of microorganisms (bacteria) found on fish [32].

### 6.3. Future Direction of Traceability

Ensuring traceability through the food chain can be accomplished by careful planning, taking the time to gain consensus among the food operators, and gaining the trust of the consumers. In order to gain consumer trust in the traceability system as a whole, the traceability system in place must meet the set standards.

To have a common understanding about food traceability among food business operators is imperative when agreeing to the introduction of a system through the food chain. Equally as important is ensuring the consistency of the food traceability system implemented by each food operator and making effective connections among all the systems.

Molecular studies will continue to clarify the taxonomic status of seafood-borne pathogens and their relationship to one another. Outbreaks of certain human pathogenic bacteria, such as members of the genus *Vibrio*, are frequently associated with the consumption of raw and undercooked shellfish [33].

## 7. Conclusions and Recommendations

### 7.1. Conclusions

- Though Baro-Akobo basin has the most diverse fish species, it contributes little to the national fish demands.
- The major constraints that reduce fish production in the Gambella region include inefficient fishing gear, poor transportation access, lack of fish handling facilities, and poor postharvest handling.
- Drying is the predominant postharvest handling technique practiced in the region.
- Major marketing constraints include low prices at the landing site, poor road access, high transportation cost, improper market place, lack of cold storage, and poor power supply.
- The fishing methods used in the study area are predominantly traditional and of a subsistence basis.
- All the fishing activities in the region take place in the natural environment, and aquaculture is not yet established.
- Insufficient institutional and management capacity, limited resource allocation and investment, poor policy and regulatory framework, and insufficient value chain and fish marketing infrastructure are some of the cross-sectoral challenges affecting fisheries in Ethiopia, particularly the Gambella region.
- Traceability is a concept utilizing a variety of tools and advanced technologies in assuring public health by inspecting food before being used.
- The most important benefit of traceability is the improvement of supply chain management. Other benefits include an increase in the ability to retain existing customers, product quality improvement, product differentiation, and reduction of customer complaints.

### 7.2. Recommendations

- Enough modern and efficient gears need to be made available in the regions to be used by the fishers
- Enough power supply needs to be installed in the Fishery Production and Marketing Enterprise collection centers and other postharvest handling techniques need to be introduced in the region to ensure a longer shelf life of fish after harvest
- Regional governments need to construct infrastructures to access all water bodies in the region and stabilize the current security problem faced by the fishers
- Because the region has such water resource potential and incredible fish species diversity, aquaculture needs to be established
- Traceability needs to be adopted and effectively implemented worldwide, including developing nations to ensure the safety of the public

## Figures and Tables

**Table 1:** Commercially important fish species in the Gambella region [14].

No.	Genera	Scientific name	Local (anywa) name	Local (nuer) name	Common name (English)
1	*Citharinus*	*C. citharus*; *C. latus*	Abel	P1t-p1t	
2	*Malapterurus*	*M. electricus*	Adaenga/Adinga	D“r/D`nvy	Electric fish
3	*Clarias*	*C. gariepinus*	Aguwella	P0t	Catfish
4	*Mormyrus*	*M. kannume*; *M. niloticus*	Dolo	Kusth	Elephant-snout fish
5	*Mormyrops*	*M. anguilloides*	Dolo	Nst-ri3l	Cornish Jack
6	*Hippopotamyrus*	*H. harringtoni*	Dolo/Auaeyt	Kusth	
7	*Lates*	*L. niloticus*	Gur	C`l	Nile perch
8	*Distichodus*;	*D. niloticus*	Puro	Yaath	
9	*Bagrus*	*B. docmac*; *B. bajad*	Udoora/Adwera	L1m	
10	*Polypterus*	*P. bichir*	Uduwella/Udeela	Ju3th	
11	*Synodontis*	*S. frontosus*; *S. clarias*, etc.	Ukok	Yivw/Xssk	
12	*Labeo*	*L. horie*	Ukura	Luabu]y/Minydh7t	
13	*Barbus*	*Barbus* spp.	Ukura	Luabu]y/Minydh7t	
14	*Auchenoglanis*	*A. occidentalis*	Ukul	Tut-d66 l	
15	*Heterotis*	*H. niloticus*	Uluak/Uloek	Lvk	
16	*Oreochromis*	*O. niloticus*	Uredo	Ru3th	Nile tilapia
17	*Sarotherodon*	*S. galilaeus*	Uredo	Burjilv/Ru3th	Tilapia
18	*Gymnarchus*	*G. niloticus*	Wit/Uit	Ri3l	Aba
19	*Hydrocynus*	*H. forskahlii*	Weari	J7k-lvc	Tiger fish
